# Integrating bilingual musculoskeletal imaging education into radiology and diagnostic imaging residency programs

**DOI:** 10.1590/0100-3984.2017.0027

**Published:** 2018

**Authors:** Francisco Abaeté Chagas-Neto, Barbara Caracas, Idalia Fortaleza, Esio Fortaleza, Eduardo Lima Rocha, Atul Kumar Taneja, Evandro Abreu, Marcello Henrique Nogueira-Barbosa

**Affiliations:** 1 MD, PhD, Coordinator of the Musculoskeletal Imaging Sector of Hospital Antônio Prudente, Professor at the Centro Universitário Christus, Fortaleza, CE, Brazil.; 2 MD, Resident at Hospital Antônio Prudente, Fortaleza, CE, Brazil.; 3 MD, Radiologist, Coordinator of the Radiology Department of Hospital Antônio Prudente, Fortaleza, CE, Brazil.; 4 MD, PhD, Radiologist at the Musculoskeletal Imaging Division of Hospital Israelita Albert Einstein, Hospital do Coração (HCor), and Teleimagem, São Paulo, SP, Brazil.; 5 Professor of Medical English at Hospital Antônio Prudente, Fortaleza, CE, Brazil.; 6 Tenured Associate Professor of Radiology, Faculdade de Medicina de Ribeirão Preto da Universidade de São Paulo (FMRP-USP), Ribeirão Preto, SP, Brazil.

**Keywords:** Multilingualism, Education, medical, Radiology, Musculoskeletal system/diagnostic imaging

## Abstract

**Objective:**

To analyze the perception of the relevance of introducing bilingual (Portuguese-English) musculoskeletal imaging education into radiology and diagnostic imaging residency programs, describing the method used.

**Materials and Methods:**

To evaluate the relevance of incorporating the bilingual study of musculoskeletal imaging into medical residency programs, we administered a questionnaire, consisting of several multiple-choice questions and one subjective question, to 21 radiology residents at a private tertiary hospital. The residents completed the questionnaire voluntarily and anonymously.

**Results:**

Integrating English teaching into radiology training was considered important by 95.2% of the residents. Approximately 90% of residents believe that the method applied at their institution is suitable for learning.

**Conclusion:**

The introduction of the English language into the teaching of musculoskeletal imaging in the radiology residency program was perceived positively by the residents, and the preceptors effectively supported those activities.

## INTRODUCTION

The need to update and improve medical professionals is constant, due to the daily rate of new medical discoveries. Since English is the major language in medical sciences^(^^1^^)^, the globalization of communication reflects the need to incorporate bilingual (in this case Portuguese-English) into radiology residency programs to enable radiologists to monitor the progress in their speciality, as well as to participate in scientific events and take advantage of other international opportunities^(^^1^^)^.

Currently, bilingual study for medical students is a routine method in several countries around the world, notably in China, where policies were implemented in 2001 to achieve that objective^(^^2^^)^. Japan has also inserted English into many areas of study, and in 2003 presented guidelines to improve the quality of English instruction for professional training, thereby better preparing their graduates for the labor market^(^^3^^)^.

There are few studies on bilingual education in medical radiology, especially in musculoskeletal imaging. Therefore, this article aims to evaluate the perception of the relevance of integrating English instruction into the training of radiology residents, as well as to describe and discuss the available literature on other bilingual education initiatives.

## MATERIALS AND METHODS

This study, conducted between February 2015 and February 2016, involved radiology residents at a private tertiary hospital in Fortaleza, a Brazilian city in the state of Ceará. During the study period, classroom sessions, case studies, and presentations of articles were conducted in English on a weekly basis. The presentations were organized in a manner that the first presentation of the month was performed by a trained attending in musculoskeletal radiology residency program (preceptor) or by the medical English professor of the institution. The remainder classes during the month were presented by residents or fellows.

The first presentation of each month (that given by a preceptor or professor) had as main goal the demonstration to residents how to create a well-developed presentation, providing models, as well as to address the questions and difficulties that are more common in the group, such as pronunciation and the ability to discuss the content in the foreign language. Recent articles on relevant musculoskeletal imaging themes, published in the main international journals, were used as reference material.

In the following weeks of each month, first- to fourth-year residents and fellows produced lectures totally in English, initially presented only to the professor of medical English, who provided guidance and corrections of pronunciation, as well as of the written content. After correction, the material was presented to the whole group. At the end, the preceptor of the musculoskeletal radiology residency program made comments on the topic. After each presentation, the English teacher provided a one-to-one feedback to the resident who taught the class, to avoid personal embarrassment or insecurity.

After the above-described teaching procedure had been employed for one year, a questionnaire was given to 21 resident physicians, to find out their opinions. The residents completed the questionnaire voluntarily and anonymously. The questionnaire included the following inquiries:

Do you consider a relevant issue to integrate English into your radiology instruction?

- How do you evaluate this method of radiology and English in the residency program?
- How do you evaluate your performance in this integrated teaching activity?
- To what degree are the preceptors, in general, committed to this activity?
- Were the chosen teaching methods adequate for learning?
- Do you have any comments or suggestions in regards to the teaching activity?

With the exemption of the final question, which allowed subjective answers, the questionnaire comprised multiple choice questions, with the following possible responses: yes or no; excellent, good, satisfactory, or poor; and completely agree, partially agree, partially disagree, or completely disagree.

## RESULTS

All 21 of the first- to fourth-year resident physicians or fellow volunteers completed the forms. Of the total (21), 20 (95.2%) considered important to integrate English into radiology instruction, whereas one (4.8%) did not.

Nineteen (90.5%) of the volunteers agreed that the method applied for bilingual instruction in musculoskeletal imaging is adequate for learning and only two (9.5%) disagreed. Neither of them disagreed completely. Nine volunteers (42.9%) evaluated their performance in this integrated bilingual education activity as good or excellent, eleven (52.4%) evaluated as satisfactory, and only one (4.8%) evaluated as poor. Regarding the statement if the preceptors were dedicated and committed to this activity, 20 volunteers (95.2%) agreed, only one (4.8%) disagreed partially.

The final question was optional and solicited observations or suggestions from the residents about this teaching activity. One participant submitted the following comments: "I believe that bilingual study is relevant but should not be exclusively bilingual. Studies in English should be intercalated with studies in the students' mother tongue. Thus, we would have a theoretical basis for more effective learning." Another volunteer commented, "I consider the integration of English into musculoskeletal radiology instruction important because it allows the resident to stay updated through reading international articles in areas where there is a greater demand."

## DISCUSSION

English proficiency is crucial for the professional growth of physicians, since it allows adequate access to updated medical information^(^^4^^,^^5^^)^, as well as participation in international conferences and meetings. In this context, the main radiology journal in Brazil accepts and had published articles in English^(^^6^^-^^12^^)^. Given the lack of studies on bilingual education in radiology, especially musculoskeletal imaging, we were motivated to analyze the integration method of English into radiology instruction.

In order to better train professionals for their career, the Japanese Society for Medical Education developed guidelines aimed at proficiency in reading and understanding books and articles in English, as well as the ability to use English in conducting medical interviews and making presentations at scientific meetings^(^^13^^)^.

English for a specific purpose (ESP) has been gaining considerable prominence in recent decades for the teaching of English. The ESP method is based on the analysis of the real needs of each student, from their level of knowledge of the language to the learning methods in which they have already exposed, along with their motivations and aspirations^(^^1^^)^. Teachers can use survey questionnaires, field observations, and interviews for such analyses and thus understand what type of learning barriers their students confront^(^^2^^)^.

Teaching English for medical purposes is based on the idea of a practical perspective. The goal is to prepare students for specific situations they will encounter in their work environment. The ESP method is focused on the context of language, along with the acquisition of specific vocabulary, giving impetus to train students in specific fields^(^^1^^)^. This method advocates the inclusion of three elements of language in the medical English classes: functional language, which refers to specific medical skills, such as dialogues used in a consultation or in the discussion of clinical cases; linguistic competence, linked to the ability to understand the language and express verbal fluency; and the system of language, directed to words that express sympathy with the patient and are of extreme importance in professional practice^(^^14^^)^.

In our study, we used scientific articles, classes, and clinical cases, simulating real-life situations and thereby promoting familiarity not only with medical vocabulary, especially in the area of musculoskeletal imaging, but also with formal vocabulary. The command of English was categorized as basic (fewer than two years of previous study) in 10% of the residents, intermediate (approximately four years of previous study) in approximately 70%, and advanced (six or more years of previous study) in 20%. We also emphasize that the only resident who felt that the integration of instruction in English into the curriculum was irrelevant was one of those with only the most basic knowledge of the language. That same resident was also the only one who reported having performed poorly on the activity, as well as being the only one to disagree (although partially) with the statement that the preceptors were dedicated and committed to the activity. We also found that the two respondents who partially disagreed with the method employed were the ones with a more basic knowledge of English. Analyzing this information, we can infer that a lack of previous knowledge of English can be an obstacle, limiting resident adherence to the method employed, and requiring personalized attention to each case to maintain their motivation and participation in the activity. In our study, we arranged, with the medical English professor, a distinguished attention and guidance schedule for the residents who demonstrated more difficulties in preparing their lectures in English.

We emphasize that the method adopted here is easily applicable to residents with intermediate or advanced English language skills, who accounted for approximately 90% of the sample. Over the course of the year, the progress in performance of the residents and their their presentations in English was evident. In general, they were motivated and confident. Even when making minor errors in pronunciation or syntax, they were able to adequately convey the message of their presentations, promoting good understanding among the listeners.

One of the most important strategies to better understand the English language is the formation of small study groups, since they allow the customization of the study approach for each student^(^^1^^)^. Our groups had an average of 15 people per presentation, thereby keeping the residents free to expose their difficulties and articulate their questions. The professor of medical English, in addition to having a good knowledge of the language, must be have a good communication and should also be familiar with the main technical terms and medical nomenclature used in the specialty, although a medical degree is not a prerequisite^(^^14^^)^.

The professor of medical English at our facility is course-certified by the leading language schools in Brazil and is the coordinator of a language center specializing in physicians and other health professionals. The internet has become a great ally in this learning experience. The easy access to websites that provide a phonetic query for each word and the possibility of hearing the correct pronunciation facilitates understanding and memorization^(^^4^^)^.

The contemporary method of bilingual education for the medical field, in most countries, is carried out using problem-solving strategies and simulations of real-life situations. This method contributes to the training of professionals with greater confidence and ability to cope with the adversities they will encounter throughout their careers. Radiology residents and fellows also benefit from the implementation of the bilingual study of musculoskeletal imaging, since this activity provides them with new sources of knowledge and makes them better prepared to work in their chosen specialty.

We conclude, based on a review of the literature and our experience, that the integration of bilingual education into radiology and diagnostic imaging residency programs is useful for training skilled professionals, enabling those professionals to perform their functions in a confident and up-to-date manner. The introduction of the English language into the teaching of musculoskeletal imaging in radiology residency was positively evaluated by the physicians surveyed in this study, and their preceptors effectively supported the activities.
